# Synthesis and Anticonvulsant Activity of Various Mannich and Schiff Bases of 1,5-Benzodiazepines

**DOI:** 10.1155/2012/237965

**Published:** 2012-11-28

**Authors:** Surendra N. Pandeya, Neha Rajput

**Affiliations:** Department of Pharmaceutical Sciences, Saroj Institute of Technology and Management, Sultanpur Road, Lucknow 226002, India

## Abstract

Benzodiazepines have a various behavioral effects in addition to their anxiolytic action. There is every reason to believe that the BZ/GABA receptor complex is involved in these effects, since GABAmimetic manipulations modify the effect of BZ in tests of convulsive activity, motor function, and appetitive behavior. 1,5-Benzodiazepines are biologically important molecules and are extensively used clinically as analgesic, hypnotic, sedative, and antidepressive agents. Hence, 1,5-Benzodiazepines were synthesized by condensation of o-phenylenediamine and ketones, for example, cyclohexanone and acetone in presence of sulfated zirconia (catalyst). Mannich bases were synthesized with acetophenone, p-nitroacetophenone, p-chloroacetophenone, and formaldehyde. Schiff bases were synthesized using Mannich base of 1,5-benzodiazepines with p-chloroaniline and p-chlorophenylsemicarbazide in the presence of glacial acetic acid. All the synthesized compounds were characterized by ^1^H NMR and IR spectral analyses. All the synthesized derivatives were evaluated at the dose of 30 mg/kg b.w for anticonvulsant activity by isoniazid induced convulsion model, and the compounds NBZD-3 and NBZD-8 were found to be the most active among all compounds. Among all the synthesized derivatives, compounds NBZD-13 and NBZD-17 were found to be the most active among all compounds using thiosemicarbazide induced model. Although NBZD-8, NBZD-10, and NBZD-18 are the compounds which had shown good anticonvulsant activity and have an advantage over that, they were not sedative.

## 1. Introduction

A benzodiazepine is a psychoactive drug whose core chemical structure is the fusion of a benzene ring and a diazepine ring. The first benzodiazepine, chlordiazepoxide (Librium), discovered accidentally by Leo Sternbach in 1955 and made available in 1960 by Hoffmann La Roche, which has also marketed diazepam (Valium) since 1963 [1]. 1,5-Benzodiazepines constitute an important class of psychopharmaca [2], in particular as tranquilizers and also as potent Virucides and nonnucleoside inhibitors of HIV-1 reverse transcriptase [3].

Benzodiazepine has a traditional place in antiepileptic therapy. The clinical use of BZDs can be divided into two categories. First, in the acute treatment of seizures as drugs of choice in status epilepticus and also in some cases of febrile seizures. Second, the BZDs are utilized in long-term therapy of certain seizures types primarily in the pediatrics' population [4]. 

There are some differences between the effects of 1,5- and 1,4-benzodiazepines. A greater therapeutic potential and lower incidence of side effects were described for 1,5-BZDs when compared to 1,4-BZDs. 1,5-BZD is used as adjuvant therapy in resistant cases of epilepsies [5]. BZDs exhibit potent anticonvulsant actions in a wide variety of animal seizures models. They are particularly effective against seizures induced by electroshock [6], and various chemoconvulsants, in kindled seizures and in absence seizures [7]. 

Beside this, 1,5-benzodiazepines show antifungal, antibacterial [8], antifeedant [9], anti-inflammatory analgesic [10], and anticonvulsant activities [11]. The benzodiazepines nucleus is a well-studied traditional pharmacophoric scaffold that has emerged as a core structure unit of various biological activities [12].

Although, the first benzodiazepine was introduced as a drug nearly 35 years ago, the research in this area is still very active and is directed towards the synthesis of compounds with enhanced pharmacological activity [13]. The chemical structure of the benzodiazepines seems at first sight to be unique among the various types of central depressant drugs [14]. 1,5-Benzodiazepines derivatives show a large number of pharmacological properties such as they acted as sedatives [15], Cerebrovasodialators [16], neuroleptics [17], antispasmodic [18], anticonvulsant [19], tranquilizing agents [20], antibacterial [21], and psoriasis [22] and used for the treatment of small pox [23].

## 2. Experimental Procedure

Starting material and reagents were procured from commercial chemical suppliers. All the chemicals and solvents used were of laboratory grade. Melting points were determined in open capillary tubes and are uncorrected. IR spectra (KBr, cm^−1^) were recorded on Perkin Elmer Spectrometer, 1H NMR-(*δ*, ppm) spectra were recorded on a Brucker 300 MHz NMR spectrometer using TMS as an internal standard. The purity of compounds and progress of the reaction were checked by TLC using silica gel-G as adsorbent. 

### 2.1. Procedure for Preparation of Fused Ring Benzodiazepine Nucleus

#### 2.1.1. Synthesis of Fused Ring Benzodiazepine Nucleus

Synthesis of fused ring benzodiazepines in presence of sulfated zirconia involves 2 steps which are as follows [9].

**Preparation of Catalyst**

25 gm of zirconium oxychloride was dissolved in doubly distilled water (pH-2). Dilute aq. ammonia was then added dropwise from a burette with vigorous (pH = 8). Precipitate was washed with distilled water several times and dried for 24 h. Sample was ground to fine powder and immersed in an 0.5 M H2SO4 solution (30 mL) for 30 min. Excess water was evaporated on water bath, and the resulting sample was oven dried.

**Synthesis of Benzodiazepines**

**Scheme 1:**
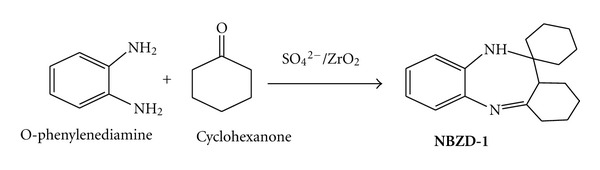
Synthesis of fused ring benzodiazepine.

1 : 2.5 mole ratio mixture of o-phenylenediamine and ketone (cyclohexanone (Scheme 1)) with catalytic amount of sulfated zirconia was taken in round bottom flask (RBF) with stirring at ambient condition for 2-3 h. 10 mL of CH2Cl2 was added to reaction mixture, and catalyst was recovered by filtration.

### 2.2. Procedure for Preparation of Mannich Base Derivatives

#### 2.2.1. Synthesis of Various Mannich Base Derivatives of Fused Ring Benzodiazepine (Scheme 2)

**Scheme 2:**
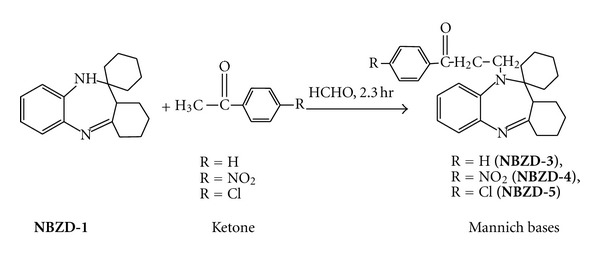
Synthesis of various Mannich base derivatives of fused ring benzodiazepines.

Equimolar quantity of fused ring benzodiazepine (**NBZD-1**, 0.01 M), formaldehyde, and various acetophenones (i.e., acetophenone, p-nitroacetophenone, and p-chloroacetophenone) were taken in RBF, and mixture was refluxed for 2.30 h. Completion of reaction was monitored by TLC analysis for several times. Then, reaction mixture was evaporated on water bath and dried. Melting point, Rf value, and % yield were noted. Various Mannich base derivatives are shown in Scheme 2.

### 2.3. Procedure for Preparation of Schiff Base Derivatives

#### 2.3.1. Synthesis of Various Schiff Base Derivatives of Fused Ring Benzodiazepines

**Scheme 3:**
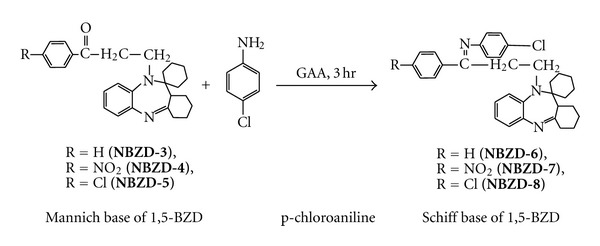
Synthesis of Schiff base derivative of fused ring benzodiazepine from p-chloroaniline.

**Scheme 4:**
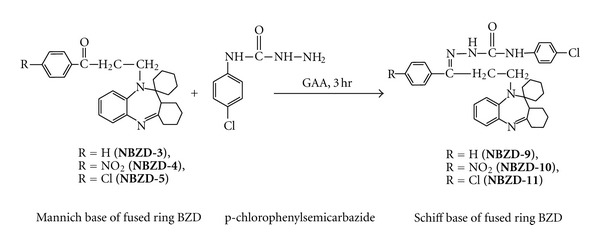
Synthesis of Schiff base derivative of fused ring benzodiazepine from p-chlorophenylsemicarbazide.

Equimolar quantities of Mannich base derivatives (0.01 M, **NBZD-3**, **NBZD-4**, and **NBZD-5**), in individual reactions, were dissolved in glacial acetic acid and added with p-chloroaniline (Scheme 3) or p-chlorophenylsemicarbazide (Scheme 4) and taken in RBF and mixture was refluxed for 3 h, respectively. Completion of reaction was monitored by TLC analysis for several times in chloroform : ethanol (1 : 1). Then, reaction mixture was evaporated on water bath and dried. Melting point, Rf value, and % yield were noted.

### 2.4. Procedure for Preparation of 1,5-Benzodiazepine Nucleus

#### 2.4.1. Synthesis of 1,5-Benzodiazepine Nucleus

Synthesis of 1,5-benzodiazepines in presence of sulphated zirconia involves 2 steps which are as follows [9].

**Synthesis of Benzodiazepines**

**Scheme 5:**
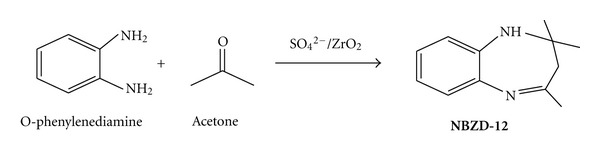
Synthesis of 1,5-benzodiazepine (**NBZD-12**).

1 : 2.5 mole ratio mixture of o-phenylenediamine and acetone (Scheme 5) with catalytic amount of sulfated zirconia was taken in round bottom flask (RBF) with stirring at ambient condition for 2-3 h. 10 mL of CH2Cl2 was added to reaction mixture, and catalyst was recovered by filtration.

### 2.5. Procedure for Preparation of Mannich Base Derivatives

#### 2.5.1. Synthesis of Various Mannich Base Derivatives of 1,5-Benzodiazepine (Scheme 6)

**Scheme 6:**
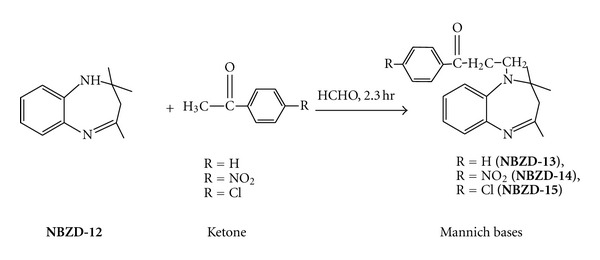
Synthesis of various Mannich base derivatives of 1,5-benzodiazepines.

Equimolar quantities of fused ring benzodiazepine (**NBZD-12**, 0.01 M), formaldehyde, and various acetophenones (i.e., acetophenone, p-nitroacetophenone, and p-chloroacetophenone) were taken in RBF, and mixture was refluxed for 2.30 h. Completion of reaction was monitored by TLC analysis for several times. Then, reaction mixture was evaporated on water bath and dried. Melting point, Rf value, and % yield were noted. Various Mannich base derivatives are shown in Scheme 6.

### 2.6. Procedure for Preparation of Schiff Base Derivatives

#### 2.6.1. Synthesis of Various Schiff Base Derivatives of 1,5-Benzodiazepines

**Scheme 7:**
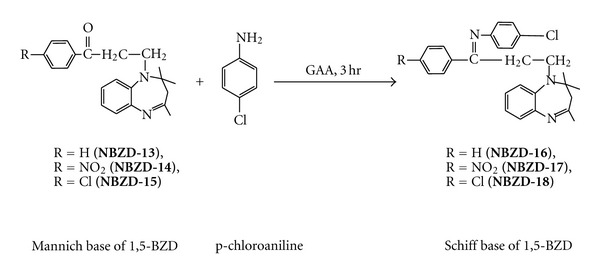
Synthesis of Schiff base derivative of 1,5-benzodiazepine from p-chloroaniline.

**Scheme 8:**
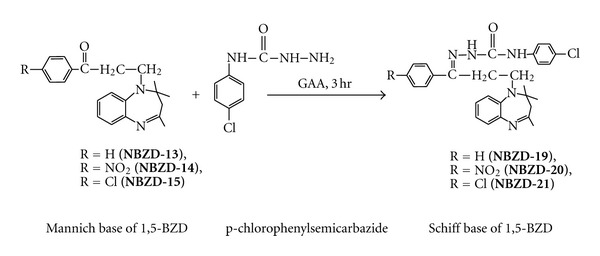
Synthesis of Schiff base derivative of 1,5-benzodiazepine from p-chlorophenylsemicarbazide.

Equimolar quantities of Mannich base derivatives (0.01 M, **NBZD-13, NBZD-14,** and** NBZD-15**),in individual reactions, were dissolved in glacial acetic acid and added with p-chloroaniline (Scheme 7) or p-chlorophenylsemicarbazide (Scheme 8), and were taken in RBF, and mixture was refluxed for 3 h, respectively. Completion of reaction was monitored by TLC analysis for several times in chloroform : ethanol (1 : 1). Then, reaction mixture was evaporated on water bath and dried. Melting point, Rf value, and % yield were noted.

## 3. Anticonvulsant Activity

### 3.1. Chemical Induced Model

Ten mice of either sex with a weight of 22–25 g were treated with the test compounds (30 mg/kg b.w) or the standard (e.g., diazepam 10 mg/kg b.w) by i.p. administration. Controls received the vehicle only. 30 min after i.p. treatment, the animals were injected with a subcutaneous dose of (300 mg/kg, s.c) isoniazid, thiosemicarbazide (20 mg/kg, s.c). The occurrence of clonic seizures, tonic seizures, and death or recovery were recorded after 0.5 hr, 1 hr, 2 hr, and 4 hr, respectively, for isoniazid induced convulsion (Table 3) and also Thiosemicarbazide induced convulsion (Table 4).

### 3.2. Neurotoxicity Screen

Minimal motor impairment was measured in mice by the rotarod test. The mice were trained to stay on an accelerating rotarod that rotates at 20 revolutions per minute. The rod diameter was 3.2 cm. Neurotoxicity was indicated by the inability of the animal to maintain equilibrium on the rod for at least 1 min in each of the three trials. The dose at which the animals were unable to grasp the rotarod was determined (Tables 3 and 4).

DOSE: Test drug: 30 mg/Kg b.w i.p. 

### 3.3. Sedative-Hypnotic Activity

This test was performed with the test substances in a dose of 30 mg/kg by phenobarbitone induced narcosis in rats. The compounds in PEG (polyethylene glycol) were administered i.p to a group of six rats. After 30 min, rats were then placed on their back and loss of righting reflex was taken as onset of sleep. The time taken by the rats to awake was noted. A control was also performed after pretreatment with test substances vehicle (PEG) and injected phenobarbitone (Table 5).

## 4. Results

### 4.1. Physicochemical Characterization

See Table 1.

### 4.2. Elemental Analysis

See Table 2.

### 4.3. Representative Spectral Analysis

1. 10-Spirocyclohexane-1,2,3,9,10,10a-hexahydrobenzo[b]cyclohexane [e][1,4] diazepine (**NBZD-1**):
2. ^1^H NMR (300 MHz, *δ*): CH(m, 6.4–7.0, 4H, phenyl), NH(s, 4.1, 1H), CH_2_(m, 1.2–1.6, 18H, cyclohexane), CH(s, 2.7, 1H, diazepine ring).
3. IR (KBr): NH(Ar, 3030 cm^−1^, str), CH(Ar, 3180 cm^−1^, str), CH(Ar, 800 cm^−1^, bend), C=N(1618 cm^−1^, Str) CH_2_(1490 cm^−1^, str), C–C(Ar, 1600 cm^−1^), C=C(Ar, 1410, 1500, 1580 cm^−1^).
4. 1-Phenyl-3-(10-spirocyclohexane-1,2,3,9,10,10a-hexahydrobenzo[b]cyclohexane [e][1,4]diazepine-1-yl) propan-1-one (**NBZD-3**):
5. ^1^H NMR (300 MHz, *δ*): CH(m, 7.3–7.9, 5H, acetophenone), CH_2_(s, 2.8, 2H, –COCH_2_), CH_2_(s, 3.5, 2H, –NHCH_2_), CH(m, 6.4–7.0, 4H, phenyl), CH_2_(m, 1.2–1.5, 18H, cyclohexane), CH(s, 2.5, 1H, diazepine ring).
6. IR (KBr): C=O(1700 cm^−1^, str), CH(Ar, 3180 cm^−1^, str), CH(Ar, 810 cm^−1^, bend), C=N(1618 cm^−1^, Str), CH_2_(1490 cm^−1^, str), C–C(Ar, 1600 cm^−1^), C=C(Ar, 1410, 1500, 1580 cm^−1^).
7. 1-(4-Nitrophenyl)-3-(10-spirocyclohexane-1,2,3,9,10,10a-hexahydrobenzo[b]cyclohexane [e][1,4]diazepine-1-yl) propan-1-one (**NBZD-4**).
8. ^1^H NMR (300 MHz, *δ*): CH(m, 8.1-8.2, 4H, p-nitroacetophenone), CH_2_(s, 2.7, 2H, –COCH_2_), CH_2_(s, 3.5, 2H, –NHCH_2_), CH(m, 6.4–7.0, 4H, phenyl), NH(s, 4.0, 1H,), CH_2_(m, 1.22–1.59, 18H, cyclohexane), CH(s, 2.7, 1H, diazepine ring).
9. IR (KBr): C=O(1710 cm^−1^, str), CH(Ar, 3150 cm^−1^, str), CH(Ar, 800 cm^−1^, bend), C=N(1658 cm^−1^, Str), CH_2_ (1490 cm^−1^, str), C–C(Ar, 1610 cm^−1^), C=C(Ar, 1410, 1560, 1580 cm^−1^), N–O(1350 cm^−1^, str).
10. (4-Chloro-phenyl)-[1-chlorophenyl-3-10-spirocyclohexane-1,2,3,9,10,10a-hexahydrobenzo[b]cyclohexane [e][1,4]diazepine-1yl)-propylidene]-amine (**NBZD-8**).
11. ^1^H NMR (300 MHz, *δ*): CH(m, 7.30–7.5, 4H, p-chloroacetophenone), CH(m, 7.2-7.3, 4H, p-chloroaniline), CH_2_(s, 1.6, 2H, –COCH_2_), CH_2_(s, 3.4, 2H, –NHCH_2_), CH(m, 6.6–7.1, 4H, phenyl), CH_2_(m, 1.3–1.5, 18H, cyclohexane), CH(s, 2.7, 1H, diazepine ring).
12. IR (KBr): C=N(1569 cm^−1^, str), C–Cl(727 cm^−1^, str), C–Cl(760 cm^−1^), C–H(2975 cm^−1^, str assym), CH(1383.9 cm^−1^, def sym.), C–H(Ar, 3072 cm^−1^, str), CH(Ar, 3150 cm^−1^, str), CH(Ar, 860 cm^−1^, bend), C=N(1678 cm^−1^, Str) CH_2_(1490 cm^−1^, str), C–C(Ar, 1600 cm^−1^), C=C(Ar, 1410, 1500, 1580 cm^−1^).
13. (4-Chlorophenylhyrazinecarboxamide)[1-nitrophenyl-3-(10-spirocyclohexane-1,2,3,9,10,10a-hexahydrobenzo[b]cyclohexane [e][1,4] diazepine-1-yl)-propylidene]-amine (**NBZD-10**).
14. ^1^H NMR (300 MHz, *δ*): CH(m,7.9–8.2, 4H, p-nitroacetophenone), NH(s, 7.0, 1H, =NNH, p-chlorophenylsemicarbazide), NH(s, 6.0, 1H, –NHC_6_H_4_Cl, p-chlorophenylsemicarbazide), CH(m, 7.2–7.6, 4H, p-chlorophenylsemicarbazide), CH(m, 6.6–7.1, 4H, phenyl), CH_2_(m, 1.3–1.5, 18H, cyclohexane), CH(s, 2.3, 1H, diazepine ring), CH_2_(s, 1.6, 2H, –COCH_2_), CH_2_(s, 3.4, 2H, –NHCH_2_).
15. IR (KBr): C=N(1569 cm^−1^, str), C–Cl(728 cm^−1^, str), C–H(2970 cm^−1^, str assym), CH(1353.9 cm^−1^, def sym.), C–H(Ar, 3062 cm^−1^, str), CH(Ar, 3180 cm^−1^, str), CH(Ar, 810 cm^−1^, bend), C=N(1638 cm^−1^, Str) CH_2_(1490 cm^−1^, str), C–C(Ar, 1680 cm^−1^), C=C(Ar, 1410, 1500, 1580 cm^−1^), NO(1380 cm^−1^, str).
16. (4-Chlorophenylhyrazinecarboxamide)[1-nitrophenyl-3-(10-spirocyclohexane-1,2,3,9,10,10a-hexahydrobenzo[b]cyclohexane [e][1,4] diazepine-1-yl)-propylidene]-amine (**NBZD-11**):
17. ^1^H NMR (300 MHz, *δ*): CH(m,7.6-7.7, 4H, p-chloroacetophenone), NH(s, 9.0, 1H, =NNH, p-chlorophenylsemicarbazide), NH(s, 6.0, 1H, –NHC_6_H_4_Cl, p-chlorophenylsemicarbazide), CH(m, 7.2–7.6,4H, p-chlorophenylsemicarbazide), CH(m, 6.6–7.1, 4H, phenyl), CH_2_(m, 1.3–1.6, 18H, cyclohexane), CH(s, 2.7, 1H, diazepine ring), CH_2_(s, 1.6, 2H, –COCH_2_), CH_2_(s, 3.4, 2H, –NHCH_2_).
18. IR (KBr): C=N(1559 cm^−1^, str), C–Cl(787 cm^−1^, str), C–Cl(769 cm^−1^), C–H(2975 cm^−1^, str assym), CH(1353.9 cm^−1^, def sym.), C–H(Ar, 3062 cm^−1^, str), CH(Ar, 3180 cm^−1^, str), CH(Ar, 880 cm^−1^, bend), C=N(1638 cm^−1^, Str) CH_2_(1490 cm^−1^, str), C–C(Ar, 1600 cm^−1^), C=C(Ar, 1410, 1500, 1580 cm^−1^).
19. (1-Phenyl-3-(2,2,4-trimethyl-2,3-dihydrobenzo[b][1,4] diazepin-1-yl)-propan-1-one) (**NBZD-13**).
20. ^1^H NMR (300 MHz, *δ*): CH(m, 7.3–7.8, 5H, Acetophenone), CH_2_(s, 2.7, 2H, —COCH_2_), CH_2_(s, 3.5, 2H, –NHCH_2_), CH(m, 6.6–7.1, 4H, phenyl), 2xCH_3_(s, 1.28, 6H), CH_3_(s, 0.9, 3H), CH_2_(s, 2.5, 2H, diazepine ring).
21. IR (KBr): C=O(1700 cm^−1^, str), NH(Ar, 3230 cm^−1^, str), CH(Ar, 3180 cm^−1^, str), CH(Ar, 800 cm^−1^, bend), C=N(1618 cm^−1^, Str), CH_3_(2980 cm^−1^, str), C–C(Ar, 1610 cm^−1^), C=C(Ar, 1410, 1500, 1580 cm^−1^).
22. 1-(4-Chloro-phenyl)-3-(2,2,4-trimethyl-2,3-dihydrobenzo[b][1,4]diazepin-1-yl)-propan-1-one (**NBZD-15**).
23. ^1^H NMR (300 MHz, *δ*): CH(m, 7.3–7.8, 4H, p-chloroacetophenone), CH_2_(s, 2.78, 2H, —COCH_2_), CH_2_(s, 3.5, 2H, –NHCH_2_), CH(m, 6.6–7.1, 4H, phenyl), 2xCH_3_(s, 1.2, 6H), CH_3_(s,0.9, 3H), CH_2_(s, 2.4, 2H, Diazepine ring).
24. IR (KBr): C=O(1710 cm^−1^, str), NH(Ar, 3030 cm^−1^, str), CH(Ar, 3280 cm^−1^, str), CH(Ar, 800 cm^−1^, bend), C=N(1618 cm^−1^, Str), CH_3_(2990 cm^−1^, str), C–C(Ar, 1600 cm^−1^), C=C(Ar, 1410, 1500, 1580 cm^−1^), C–Cl(760 cm^−1^).
25. (4-Chloro-phenyl)-[1-(4-nitrophenyl)-3-(2,2,4-trimethyl-2,3-dihydro-benzo[b][1,4] dizepine-1-yl)-propylidene (**NBZD-17**).
26. ^1^H NMR (300 MHz, *δ*): CH(m, 7.8–8.2, 4H, p-nitroacetophenone), CH(m, 7.2-7.3, 4H, p-chloroaniline), CH_2_(s, 1.6, 2H, –N=C–CH_2_), CH_2_(s, 3.4, 2H, –NHCH_2_), CH(m, 6.6–7.1, 4H, phenyl), 2xCH_3_(s, 1.2, 6H), CH_3_(s,0.9, 3H), CH_2_(s, 2.5, 2H, diazepine ring).
27. IR (KBr): C=N(1599 cm^−1^, str), C–Cl(728 cm^−1^, str), C–H(2985 cm^−1^, str assym), CH(1353.9 cm^−1^, def sym.), C–H(Ar, 3062 cm^−1^, str), CH(Ar, 3180 cm^−1^, str), CH(Ar, 800 cm^−1^, bend), C=N(1618 cm^−1^, Str), CH_3_(2990 cm^−1^, str), C–C(Ar, 1600 cm^−1^), C=C(Ar, 1410, 1500, 1580 cm^−1^), NO(1350 cm^−1^, str)
28. 4-Chlorophenylhyrazinecarboxamide) [1-nitrophenyl-3-(2,2,4-trimethyl-2,3-dihydrobenzo[b][1,4]diazepine-1-yl)-propylidene]-amine (**NBZD-20**).
29. ^1^H NMR (300 MHz, *δ*): CH(m, 7.9–8.2, 4H, p-nitroacetophenone), NH(s, 9.0, 1H, =NNH, p-chlorophenylsemicarbazide), NH(s, 6.0, 1H, –NHC_6_H_4_Cl, p-chlorophenylsemicarbazide), CH(m, 7.2–7.5,4H, p-chlorophenylsemicarbazide), CH_2_(s, 1.6, 2H, –N=C–CH_2_), CH_2_(s, 3.4, 2H, –NHCH_2_), CH(m, 6.6–7.1, 4H, phenyl), 2xCH_3_(s, 1.28, 6H), CH_3_(s,0.9, 3H), CH_2_(s, 2.5, 2H, diazepine ring).
30. IR (KBr): C=N(1570 cm^−1^, str), C–Cl(730 cm^−1^, str), C–H(2975 cm^−1^, str assym), CH(1353.9 cm^−1^, def sym.), C–H(Ar, 3062 cm^−1^, str), CH(Ar, 3180 cm^−1^, str), CH(Ar, 800 cm^−1^, bend), C=N(1618 cm^−1^, Str), CH_3_(2990 cm^−1^, str), C–C(Ar, 1600 cm^−1^), C=C(Ar, 1410, 1500, 1580 cm^−1^), NO(1350 cm^−1^, str).

### 4.4. Anticonvulsant Activity Using Chemical Induced Method

See Tables 3 and 4.

### 4.5. Sedative Activity

See Table 5.

## 5. Conclusion 

All the synthesized derivatives were evaluated at the dose of 30 mg/kg b.w for anticonvulsant activity by isoniazid induced convulsion model and the compounds **NBZD-3** and **NBZD-8** were found to be most active among all compounds. Among all the synthesized derivatives, compounds **NBZD-13**, and **NBZD-17** were found to be most active among all compounds using thiosemicarbazide induced model. Activity of the drugs interfering with motor coordination was checked by the rotarod test. None of the synthesized compounds were found to be neurotoxic at a dose of 30 mg/kg b.w among all the tested compounds. The compounds **NBZD-1**, **NBZD-3**, **NBZD-4**, **NBZD-7**, **NBZD-11**, **NBZD-12**, **NBZD-14**, **NBZD-15**, **NBZD-17**, **NBZD-20**, **NBZD-21** were found to cause sedation. Although **NBZD-8**, **NBZD-10**, and **NBZD-18** are the compounds which had shown good anticonvulsant activity and have an advantage over that, they were not sedative. 

 Sulfated zirconia is one of the important agent that has attracted much attention recently because of its superacidity, nontoxicity, and low cost. Sulfated zirconia catalyzes many reactions under very mild condition in vapor as well as liquid phase.

## 6. Structure Activity Relationship

### 6.1. Anticonvulsant (Scheme 9)

1. Highly active: **NBZD-3**, **NBZD-8**, **NBZD-13**, and **NBZD-18**.
2. Moderately active: **NBZD-1**, **NBZD-6**, **NBZD-10**, **NBZD-11**, **NBZD-12**, and **NBZD-16**, **NBZD-21**.
3. Less active: **NBZD-4**, **NBZD-7**, **NBZD-9**, **NBZD-14**, **NBZD-15**, **NBZD-17**, **NBZD-19**, and **NBZD- 20**.

Most functional subtypes of the GABA_A_ receptor contain *α*, *β*, subunits, with the different benzodiazepine binding site ligands. BZ-binding site ligands act through mechanisms which modulate the inhibiting effects of GABA.

1. In the basic structure of benzodiazepine, early SAR studies indicated that the seven-membered imino ring B was essential for its affinity towards the BZ- binding site.
2. 4-5 Carbimino double bond has also been shown to substantially contribute to the binding affinity of compound. Saturation leads to complete less of activity. It acts as a two-electron donor site.
3. The primary chemical moieties of the compounds which contribute to high receptor binding affinity are restricted to positions 7, 2, 1.
  - Position 2 is the most effective place. Presence of an electrophilic and bulky substituent at position 2 results in strong increase in receptor binding affinity of the corresponding compounds.
  - Compounds **NBZD-3** and **NBZD-8** had shown good anticonvulsant activity as they have cyclohexane ring at position 2.
4. Molar refractivity is the most important parameter at position 1, suggesting that the molecular size of the substituent needs to be restricted at position 1 for effective ligand binding. Compounds **NBZD-1** and **NBZD-8** have less substituent as compared to other and hence more active.
5. Compounds **NBZD-6**, **NBZD-10**, and **NBZD-11** were found to be moderately active anticonvulsant action, hence this shows that chloro-substituted derivatives are rather good anticonvulsant agent as compared to nitro-substituted derivatives.
6. Among these synthesized compounds which have methyl groups at the 2nd and 4th position, **NBZD-13**, and **NBZD-18** were found to be most active. Hence it shows that good activity compounds are preferred with less substitution the at 1st position.

## Figures and Tables

**Scheme 9:**
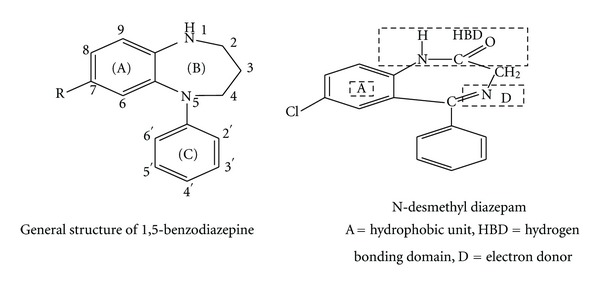


**Table 1:** Physicochemical data of synthesized compounds.

Compound code	Molecular formula	Molecular weight	Melting point (°C)	% yield	Rf value	Log *P* value
**NBZD-1**	C_18_H_24_N	268.19	110	76.48	0.689	4.25
**NBZD-3**	C _27_H_32_N_2_O	400.25	92	85.7	0.78	6.25
**NBZD-4**	C_33_H_36_N_3_Cl	510.12	132	83.3	0.73	6.15
**NBZD-5**	C_34_H_28_ClN_5_O	568.15	140	72	0.83	6.81
**NBZD-6**	C_27_H_31_N_3_O_3 _	445.55	98	84.3	0.63	8.93
**NBZD-7**	C_27_H_31_ClN_2_O	435.00	98	79.2	0.82	6.70
**NBZD-8**	C_33_H_35_ClN_4_O_2_	554.24	130	67.2	0.57	9.49
**NBZD-9**	C_34_H_37_ClN_6_O_3_	612.26	126	56.98	0.52	8.07
**NBZD-10**	C_33_H_35_Cl_2_N_3_	543.22	136	65.8	0.54	9.56
**NBZD-11**	C_34_H_37_Cl_2_N_5_O	601.24	130	66	0.66	8.62
**NBZD-12**	C_12_H_16_N_2_	188.60	96	72	0.68	2.22
**NBZD-13**	C_21_H_24_N_2_O	320.3	98	72.32	0.72	4.22
**NBZD-14**	C_21_H_23_N_3_O_3_	365.43	102	92.1	0.72	4.83
**NBZD-15**	C_21_H_24_N_2_O	320.43	110	57.6	0.75	4.78
**NBZD-16**	C_21_H_23_ClN_2_O	354.87	108	82.9	0.73	6.90
**NBZD-17**	C_27_H_28_ClN_3_	429.98	140	90.4	0.78	7.20
**NBZD-18**	C_27_H_27_ClN_4_O_2_	474.98	136	64.06	0.75	7.46
**NBZD-19**	C_27_H_27_Cl_2_N_3_	464.43	142	85.6	0.63	6.04
**NBZD-20**	C_27_H_27_ClN_4_O_2_	474.18	138	67.9	0.55	—
**NBZD-21**	C_28_H_29_ClN_6_O_3_	532.20	142	65.2	0.76	6.59

Rf value: solvent system; chloroform : methanol 1 : 1.

**Table 2:** Elemental analysis of synthesized compounds.

Compound code	Calculated value			Observed value		
	C	H	N	C	H	N
**NBZD-1**	80.55	9.01	10.44	80.31	8.90	10.24
**NBZD-3**	80.96	8.05	6.99	80.56	8.00	6.79
**NBZD-4**	72.78	7.01	9.43	72.70	6.89	9.23
**NBZD-5**	74.55	7.18	6.44	74.23	7.04	6.25
**NBZD-6**	77.70	7.11	8.24	77.56	7.01	8.16
**NBZD-7**	71.40	6.26	10.09	71.19	6.16	10.00
**NBZD-8**	72.78	6.48	7.72	72.66	6.32	7.61
**NBZD-9**	71.88	6.74	12.33	71.34	6.66	12.21
**NBZD-10**	66.60	6.08	13.71	66.45	6.00	13.62
**NBZD-11**	67.77	6.19	11.62	67.44	6.02	11.52
**NBZD-12**	76.55	8.57	14.88	76.25	8.34	14.54
**NBZD-13**	78.71	7.55	8.74	78.59	7.56	8.70
**NBZD-14**	69.02	6.34	11.50	68.66	6.30	11.43
**NBZD-15**	71.07	6.53	7.89	71.00	6.45	7.68
**NBZD-16**	75.42	6.56	9.77	75.21	6.36	9.53
**NBZD-17**	68.27	5.73	11.80	68.20	5.60	11.70
**NBZD-18**	69.83	5.83	9.05	69.70	5.78	8.90
**NBZD-19**	68.91	6.20	3.28	68.78	6.09	3.10
**NBZD-20**	63.09	5.48	15.77	63.01	5.32	15.57
**NBZD-21**	64.37	5.59	13.40	64.30	5.40	13.28

**Table 3:** Anticonvulsant activity using Isoniazid induced convulsion model.

Compound code	Isoniazid induced model			Neurotoxicity study	
	0.5 hr	1 hr	2 hr	1 hr	4 hr
				Dose: 30 mg/kg	
**NBZD-1**	30 mg	30 mg	Not protected	NN	NN
**NBZD-3**	30 mg	30 mg	30 mg	NN	NN
**NBZD-4**	30 mg	Not protected	Not protected	NN	NN
**NBZD-5**	30 mg	Not protected	Not protected	NN	NN
**NBZD-6**	30 mg	30 mg	Not protected	NN	NN
**NBZD-7**	30 mg	Not protected	Not protected	NN	NN
**NBZD-8**	30 mg	30 mg	30 mg	NN	NN
**NBZD-9**	30 mg	Not protected	Not protected	NN	NN
**NBZD-10**	30 mg	30 mg	Not protected	NN	NN
**NBZD-11**	30 mg	30 mg	Not protected	NN	NN
Control	—	—	—		

Symbol (NN) indicates no neurotoxicity at 30 mg/kg b.w.

**Table 4:** Anticonvulsant activity using thiosemicarbazide induced convulsion model.

Compound code	Thiosemicarbazide induced model			Neurotoxicity study	
	0.5 hr	1 hr	2 hr	1 hr	4 hr
				Dose: 30 mg/kg	
**NBZD-12**	30 mg	30 mg	Not protected	NN	NN
**NBZD-13**	30 mg	30 mg	30 mg	NN	NN
**NBZD-14**	30 mg	Not protected	Not protected	NN	NN
**NBZD-15**	30 mg	Not protected	Not protected	NN	NN
**NBZD-16**	30 mg	30 mg	Not protected	NN	NN
**NBZD-17**	30 mg	Not protected	Not protected	NN	NN
**NBZD-18**	30 mg	30 mg	30 mg	NN	NN
**NBZD-19**	30 mg	Not protected	Not protected	NN	NN
**NBZD-20**	30 mg	Not protected	Not protected	NN	NN
**NBZD-21**	30 mg	30 mg	Not protected	NN	NN
Control	—	—	—		

Symbol (NN) indicates no neurotoxicity 30 mg/kg of b.w.

**Table 5:** Sedative activity of synthesized compound.

Compounds code	Sleeping time (mean ± SEM) (min)
**NBZD-1**	120 ± 9.00∗∗
**NBZD-3**	138 ± 10.53∗∗
**NBZD-4**	140 ± 11.92∗∗
**NBZD-7**	141 ± 11.21∗∗
**NBZD-8**	68 ± 12.6 NS
**NBZD-10**	63 ± 9.05 NS
**NBZD-11**	148 ± 12.15∗∗
**NBZD-12**	124 ± 10.12∗∗
**NBZD-14**	110 ± 11.41 ∗∗
**NBZD-15**	100 ± 10.98∗∗
**NBZD-17**	148 ± 11.54∗∗
**NBZD-18**	76 ± 10.26 NS
**NBZD-20**	112 ± 9.62∗∗
**NBZD-21**	157 ± 12.09∗∗
Phenobarbitone (control )	56 ± 11.47

Values represent the mean ± SEM of six animals for each group.

*Significant at *P* < 0.05 and ∗∗significant at *P* < 0.01 (Dunnett's test).

Test drug (30 mg/kg) and Phenobarbitone (40 mg/kg).

NS denotes not significant at *P* < 0.01 (student's *t*-test).
